# Cognitive bias modification for facial interpretation: a randomized controlled trial of transfer to self-report and cognitive measures in a healthy sample

**DOI:** 10.1098/rsos.170681

**Published:** 2017-12-13

**Authors:** S. E. Peters, J. Lumsden, O. H. Peh, I. S. Penton-Voak, M. R. Munafò, O. J. Robinson

**Affiliations:** 1 Institute of Cognitive Neuroscience, University College London, London, UK; 2 School of Experimental Psychology, University of Bristol, Bristol, UK; 3 MRC Integrative Epidemiology Unit at the University of Bristol, Bristol, UK

**Keywords:** facial interpretation, cognitive bias modification, translational research, randomized controlled trial

## Abstract

Cognitive bias modification is a potential low-intensity intervention for mood disorders, but previous studies have shown mixed success. This study explored whether facial interpretation bias modification (FIBM), a similar paradigm designed to shift emotional interpretation (and/or perception) of faces would transfer to: (i) self-reported symptoms and (ii) a battery of cognitive tasks. In a preregistered, double-blind randomized controlled trial, healthy participants received eight online sessions of FIBM (*N* = 52) or eight sham sessions (*N* = 52). While we replicate that FIBM successfully shifts ambiguous facial expression interpretation in the intervention group, this failed to transfer to the majority of self-report or cognitive measures. There was, however, weak, inconclusive evidence of transfer to a self-report measure of stress, a cognitive measure of anhedonia, and evidence that results were moderated by trait anxiety (whereby transference was greatest in those with higher baseline symptoms). We discuss the need for work in both larger and clinical samples, while urging caution that these FIBM training effects may not transfer to clinically relevant domains.

## Highlights

— Facial interpretation bias modification (FIBM) was studied.
— FIBM induced a substantial positive bias, alongside no change in a control group.
— FIBM effects did not appear to transfer to the majority of outcome measures.
— Inconclusive evidence of transfer to measures of stress response and anhedonia.
— This FIBM task may not be an efficacious intervention in affective disorders.

## 1. Introduction

Although a number of proven psychological and pharmacological treatments for mood disorders are available, worldwide, access to these treatments remains limited. In the UK, for instance, public treatment for depression is plagued by wait lists, high costs or side effects [1], and treatments indicate response rates of only around 50% [1,2]. Thus, there is a need for effective interventions that are inexpensive, quick and easy to deliver. It is here that cognitive bias modification (CBM) may hold promise. CBM generally consists of a short task that serves to train individuals to shift towards (a positive) or away from (a negative) cognitive processing bias [3]. Negative cognitive biases are associated with the tendency to attend to and interpret ambiguous or neutral information in a negative manner. Such biases are thought to be critical to the onset, maintenance and recurrence of anxiety disorders and depression (e.g. [4–13]). A meta-analysis of previous work has also argued for a relationship between negative cognitive biases and past, current and future depressive symptoms [14]. The emerging field of CBM posits that through the modification of these biases it may be possible to intervene prior to the onset of depression or prevent the risk of subsequent depressive episodes for individuals in remission [15].

A newly developed CBM task design (described here as FIBM) that targets the interpretation (and/or perception) of emotional facial expressions (happy versus sad) via an online training procedure [16] represents a promising candidate. The ability to recognize and comprehend emotion in facial expressions is critical for social functioning and is disrupted in depression (e.g. [17]), but is also relevant to the non-depressed population (e.g. interpreting ambiguous facial expressions as negative rather than positive will have a negative impact on one's mood regardless of the presence of an affective disorder; e.g. [9,10]). Although the misinterpretation of facial expressions has been shown in social anxiety [18], this FIBM task focuses on inducing a positive bias for happy versus sad faces relevant to depression (e.g. compared with happy versus threatening faces, which might be used in a comparable paradigm for anxiety). A number of studies have shown that it is possible to shift cognitive biases (and induce positive changes in mood) in both healthy (e.g. [19]) and dysphoric individuals (which often goes undiagnosed [20]). Indeed, using this FIBM task, Penton-Voak *et al*. [21] showed increased perception of happiness (versus sadness) in ambiguous facial expressions, which was retained for at least two weeks, as well as some evidence for increased positive affect in healthy adults, and Dalili *et al*. [22] showed that the task generalizes to non-trained facial stimuli. In theory, a positive bias in the perception of ambiguous emotional expressions should lead an individual towards a more favourable assessment of daily social interactions, and this positive framework (or ‘schemata’, [23]) should ultimately carry over into their mood and other areas of cognition across both healthy and patient populations (e.g. [13,24]). However, these more comprehensive ‘transfer’ effects for reducing depressive symptoms have yet to be extensively studied.

Previous studies on the therapeutic effects of CBM, which can be used to target attentional (attention bias modification, ABM) or interpretive (interpretation bias modification, IBM) cognitive biases, have been mixed. Clinical studies on depression are limited but have shown ABM to be an effective tool for reducing negative biases and the risk of recurrence in patients with remitted depression [15], and both ABM [25,26] and IBM [27,28] to reduce symptoms in individuals with sub-threshold depression. Following two weeks of ABM, Yang *et al*. [13] also reported symptom reduction in individuals with moderate–severe depression, which was maintained at three- and seven-month follow-up, and Beevers *et al*. [24] found that both placebo and active CBM lead to similar depression reductions over the course of a one-month period with some evidence of transfer effects to other cognitive processes. However, Everaert *et al*. [29] found that single-session dot-probe training similar to that used in the clinical studies did not successfully modify attentional biases or transfer to an interpretation bias task. Meta-analyses of the clinical efficacy of both ABM and IBM in depression have also failed to show clear evidence of changes in depressive symptomatology [30–33], though there is presently a much larger body of work considering ABM. Many of these reviews criticize the current body of CBM research for showing small effects of bias modification, running training over too few sessions (e.g. insufficient training ‘dose’), and neglecting to look at transfer effects. Our present study aims to address these shortfalls in assessment of our novel intervention through comprehensive, well-powered tests of transfer effects using a preregistered design that limits analytical flexibility and post hoc hypothesizing.

We had three specific aims. Firstly, we aimed to replicate findings by Penton-Voak *et al*. [21] in order to evaluate the efficacy of this FIBM task in inducing a positive cognitive bias. As outlined above, altering negative cognitive biases may be critical in treating affective disorders yet the clinical potential of CBM remains unclear. There is a long literature showing the misinterpretation of faces in depression (as well as other mood and anxiety disorders, e.g. [11,18,34]), which this FIBM task specifically targets. Moreover, this task is inexpensive, as well as quicker and easier (simple to use by virtue of only requiring a single choice, not dependent on reaction time and can be completed online) than many bias modification alternatives. Given this, we chose to assess this FIBM task in a healthy sample as proof of concept. Studies such as these are imperative for understanding a technique's potential prior to considering it in a clinical population. Secondly, we aimed to evaluate the efficacy of this FIBM task to improve subjective mood symptoms. Thirdly, we aimed to assess the ability of FIBM to transfer to a battery of cognitive tasks, including a dot-probe task commonly used in CBM studies [35], as well as validated cognitive measures of anhedonia [36] and stress reactivity [37]. The latter was adopted because it may be that cognitive biases are most prominent when the individual is under stress (e.g. [23]). If an intervention claims to have an effect on mood, it should also successfully shift to these other measures, which have also been argued to index mood state [35–37]. Our design, hypotheses and statistical analyses were registered online prior to data collection.^1^ We predicted that the double-blind randomized controlled FIBM would induce a positive bias in the intervention group, whereas there would be no change in a control (sham-FIBM) group. Critically, we also predicted that training effects would generalize across a battery of self-report and cognitive processing outcome measures.

## 2. Methods

### 2.1. Sample screening

All participants (recruited from the UCL Institute of Cognitive Neuroscience subject database) had normal or corrected-to-normal (glasses/contact lenses) colour vision, English as their first language and the ability to give written informed consent. Screening included the exclusion of individuals currently receiving treatment for depression or anxiety, with serious medical conditions, known psychiatric or neurological disorders, or recent engagement in drug/heavy alcohol use. All participants provided written informed consent before taking part in the study (UCL ethics reference: 1764/001) and were compensated £30 for their time.

### 2.2. Facial interpretation bias modification

#### 2.2.1. Task structure

**Figure 1.**
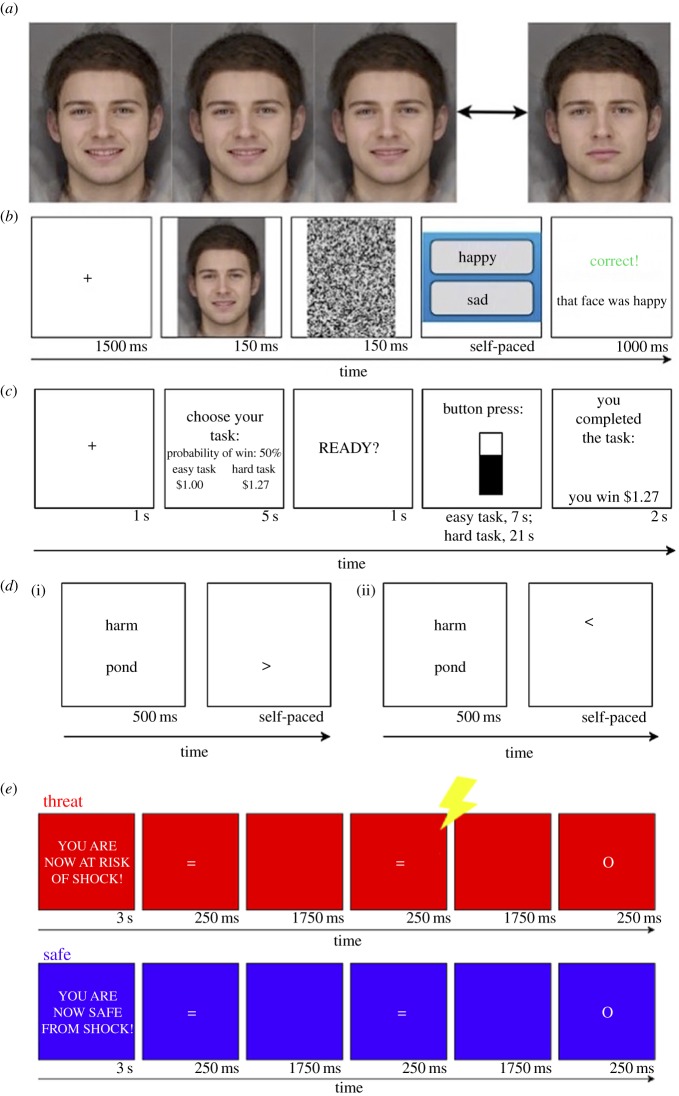
Task schematics. (*a*) Bias modification happy face morph (faces 3–5 on 15 face positive-neutral to negative-neutral spectrum) and a sad face (face 12). (*b*) Example bias modification trial with feedback (correct/incorrect window; in the training phase only). Baseline and test phases follow the identical structure but do not provide feedback. (*c*) Example of a successful hard task selection in EEfRT task. (*d*) Two words differing in emotional valence (threat/neutral) were presented simultaneously (500 ms), followed by a probe (> or <) that replaced either I. the neutral word or II. the threat word. (*e*) Participants pressed the space bar as quickly as possible in response to frequent ‘go’ stimuli (‘ = ’) and inhibited their response to infrequent ‘no-go’ stimuli (O).

Each FIBM session [16] comprised three phases (baseline, training and test), during which participants categorized facial expressions in a two-alternative forced choice procedure.^2^ In each trial participants were presented with an emotional face stimulus (1/15; figure 1*a*) which was a morph image of two emotions (happy or sad exemplars). The face pictures were morphed from several faces and were licensed from Cambridge Cognition. Participants were required to decide whether the face presented was ‘happy’ or ‘sad’ before moving to the next face.

In the baseline phase, participants did not receive feedback (they were not told if they were correct or not) in order to assess individually tailored balance points, described as the point in which they were equally likely to perceive happy or sad. Balance points were used to establish the success of the procedure in modifying the perception of ambiguous emotional expressions [21]. The baseline balance point was estimated at the end of the first block using the formula: ([number of faces categorized as happy/45] × 15), rounded to an integer. This was then used to tailor personal feedback to each participant in the training phase. In the intervention arm, participants were given feedback using a balance point shifted two places from their baseline (e.g. if their baseline balance point was 6, they were trained to 8). In the placebo arm participants were simply trained to reinforce their own balance point (e.g. if their baseline was 6, they were trained to 6). Finally, the test phase (identical to the baseline phase) measured whether training had changed the interpretation of the emotional face stimuli. The baseline and test comprised 45 trials each, where every face in the morph sequence was presented three times and in a random order. The training phase comprised 30 trials with each face presented two times (figure 1*b*). The primary outcome was the balance point at the baseline phase. The training procedure itself was identical to that used in Penton-Voak *et al*. [21].

### 2.3. Self-report outcome measures

Five self-report outcome measures were recorded. The Beck Depression Inventory II (BDI-II; [38]) measured depressive symptoms, the State-Trait Anxiety Inventory (STAI; [39]) measured state and trait anxiety, the Positive and Negative Affect Scale (PANAS; [40]) measured current affective state, the Daily Stress Inventory (DSI; [41]) assessed stress caused by daily events (e.g. ‘waited longer than you wanted’) in the past 24 h, and the Ambiguous Interpretation of Emotional Outcomes (AIEO; [42]) measured emotional interpretational biases. Outcome measures for the DSI comprised frequency, which refers to the number of events reported to have occurred in the past 24 h, and average impact rating (AIR), which refers to sum of the stress ratings attributed to these events divided by the frequency.

### 2.4. Cognitive processing outcome measures

#### 2.4.1. Effort-expenditure for rewards task

The effort-expenditure for rewards task (EEfRT) [36], which tests physical effort and motivation for hypothetical monetary rewards, was used as it has been shown to measure anhedonia [36]. In each trial, participants were given the choice between an ‘easy’ and ‘hard’ task, each of which required different amounts of fast-paced manual key pressing. The easy task required fewer key presses with the participant's dominant index finger while the hard task required more key presses with their non-dominant little finger. Participants first completed three ‘hard task’ calibrations, where they were given 21 s to make as many key presses as possible (to fill a bar on the screen). Practice and real trials that followed were calibrated and presented to all participants in the same randomized order. To successfully complete the hard task, participants were required to complete 85% of the key presses made during the calibration in 21 s while the easy task required 33% of calibrated key presses in 7 s. The (hypothetical) reward for the easy task was fixed at $1.00, while the reward for the hard task varied between $1.24 and $4.00. As monetary rewards were not guaranteed, a probability cue (12%, 50% or 88%) was given, referring to the likelihood that they would receive a reward if they completed the trial successfully. If the hard task was not chosen during the choice presentation (5 s), the easy task was automatically selected (see figure 1*c* for trial example). Participants played the task for 20 min with no maximum number of trials. The primary outcome measure for this task was the proportion of hard trials chosen.

#### 2.4.2. Emotional dot probe

This task was adopted as a common benchmark in CBM studies [32,43], as it is thought to measure differences in selective attention and response to emotional stimuli [35]. Participants were asked to identify the orientation of a non-emotional probe (> or <) from one of two spatial locations on a screen. Before the probe was presented, participants were briefly (500 ms) shown two words, which appeared simultaneously on the screen and differed in emotional valence (threat or neutral). Thirty-four pairs of threat/neutral stimuli were presented twice, once where the probe replaced the neutral word and again where it replaced the threat word, comprising 68 trials (figure 1*d* for a schematic). A threat bias score (the difference in the mean RT/accuracy to the probe following threat versus neutral stimuli priming) was calculated as the primary outcome measure.

#### 2.4.3. Stress-sustained attention to response task

This task was adopted as a reliable measure of individual stress response [37]. The use of a sustained attention task within a stress reactivity manipulation has been shown to specifically improve the ability to withhold the infrequent ‘no-go’ responses in the task (stress-SART [44]). This task was selected in order to measure whether the emotional effect of the modification was more evident when the individual encountered a stressor (e.g. [23]).

#### 2.4.4. Stress manipulation

Stress was manipulated by placing participants under threat of shock. Two electrodes were attached to the back of participants' non-dominant wrist (half a centimetre apart) with conductive gel and hypoallergenic tape. A Digitimer DS5 Constant Current Stimulator (Digitimer Ltd, Welwyn Garden City, UK) was used to generate and deliver the shocks. In order to keep the level of shocks consistent in feeling between participants, a short work-up was carried out; the level of shock was increased until participants rated it as ‘unpleasant but not painful’ [45], which took approximately three to five shocks. Threat blocks, during which participants were told they would be at risk of an unpredictable shock (non-contingent on task performance), were counterbalanced with safe blocks, where participants were told they were safe from unpredictable shock.

#### 2.4.5. Task structure

Participants were asked to press the space bar as quickly as possible (using their dominant hand) in response to frequent ‘go’ stimuli ( = ), and inhibit their response to infrequent ‘no-go’ stimuli. These stimuli were presented for 250 ms with a 1750 ms inter-stimulus interval. Eight blocks lasting 104 s were presented (alternating between threat and safe). Each block was preceded by a 3 s cue which stated either, ‘YOU ARE NOW SAFE FROM SHOCK!’ or ‘YOU ARE NOW AT RISK OF SHOCK!’. In each testing session, participants received a total of three shocks (not including those administered in the work-up); one shock was sent in the first, second and fourth threat blocks. The primary outcome measure for this task was the accuracy to ‘no-go’ stimuli (see figure 1*e* for a schematic representation).

#### 2.4.6. Retrospective state manipulation check

Immediately following the stress-SART, participants filled in a subjective manipulation check. This asked how anxious, stressed and afraid they felt in each condition (safe/threat) by selecting a number on a scale of 1 (not at all) to 10 (very much so) and ‘Did you receive a shock?’ which they responded to with ‘Yes’ or ‘No’.

### 2.5. Outcome measure programming

Questionnaires, the AIEO and the emotional dot probe, recoded for online delivery, were run online through Testable [46]. The EEfRT was run in Matlab (2014b, The MathWorks, Inc., Natick, MA, USA). The stress-SART task was run through the Cogent (Wellcome Trust Centre for Neuroimaging and Institute of Cognitive Neuroscience, UCL, London, UK) toolbox for Matlab (2014b), and the retrospective state manipulation check was run in Qualtrics (Qualtrics, Provo, UT, USA).

### 2.6. Procedure

See electronic supplementary material for a CONSORT summary of the study procedure. Following enrolment, participants were randomly allocated to either the control or intervention group according to a pre-allocated participant number. An individual (who played no role in recruitment or participant contact) randomly assigned participant identification numbers to blinded intervention logins such that group allocation was fully blind to both the participant and experimenter. Participants were asked to complete the FIBM task from a desktop computer/laptop once daily (six at-home sessions in total for which they received reminder emails at 08.00) in between in-laboratory sessions at baseline (before FIBM) and post-training (immediately after the final session of FIBM).

### 2.7. Statistical analyses

#### 2.7.1. Power calculation

*A priori* power analysis was run in G × Power [47]. This analysis was based on an effect size obtained after a six-week follow-up on FIBM training; *d* = 1.12 (M Munafò 2015, personal communication). In our determination, 50% of this value was calculated (i.e. *d* = 0.56) in order to provide a conservative estimate for the transfer effect. These data determined that we would require a sample size of 104, split evenly between intervention and control groups, to achieve 80% power at an *α* level of 5% (two-tailed).

#### 2.7.2. Frequentist analysis

Frequentist statistics were computed in SPSS v. 22 (IBM Crop, Armonk, NY, USA). For all measures, frequentist repeated measures analysis of variance (ANOVA) models were performed to compute *F*-statistics, *p*-values and effect sizes. For all analyses, our primary outcome of interest was the omnibus interaction.

#### 2.7.3. Bayesian analysis

Bayesian analyses were run in JASP [48], using the default prior, as the addition of these analyses enabled acceptance/rejection of the experimental/null hypothesis. It also enabled assessment of the relative strength of a given hypothesis. The best model for describing the data was defined as the model with the largest BF_10_. Models with a BF_10_ greater than 1 were favoured over the null model. In order to calculate whether a model including the interaction between group and time was better than a model including only time (as many measures show large repetition effects that are unrelated to our manipulation), the BF_10_ of the group × time model was divided by the BF_10_ of the time model so that resulting BF values greater than 1 favoured the group × time model. BF values were interpreted as either anecdotal (0–3), substantial (3–10), strong (10–30), very strong (30–100) or decisive (greater than 100; [49–51]). BF_10_ values below zero were taken as evidence *for* the null, with interpretation proceeding along the same lines but for the 1/BF_10_ values (e.g. 3 : 10 is substantial evidence for the model, so 1/3 : 1/10 = 0.3 : 0.1 was substantial evidence for the null). Data are available for download (https://osf.io/7uzwx/).

## 3. Results

### 3.1. Participants

We screened *N* = 111 participants, but seven were excluded due to dropout after the baseline session of the experiment (*N* = 5 in intervention group). Final participants comprised *N* = 104, of which *N* = 52 (35 female, 17 male; mean age = 24.06, s.d. = 5.13; *N* = 47 right handed) were allocated to the intervention condition and *N* = 52 (38 female, 14 male; mean age = 24.88, s.d. = 6.25, *N* *=* 49 right handed) were allocated to the control condition.

### 3.2. Facial interpretation bias modification

Within our total sample the mean baseline balance point was 7.52 (s.d. = 1.44). There were six participants with a pre-existing strong positive bias (one participant with 11 and five participants with 10 as their baseline balance point).

**Figure 2.**
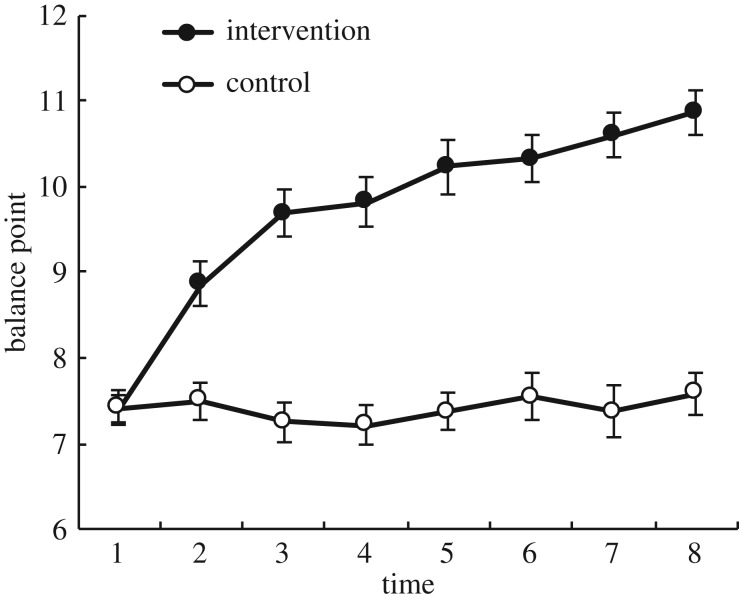
Mean baseline phase balance point for intervention and control participants across eight FIBM sessions. Error bars indicate standard error of the mean (s.e.m.).

We were able to replicate Penton-Voak *et al.*'s [21] findings; the modification successfully induced a positivity bias in the intervention group and not in the control group (time: *F* = 18.62, *p* < 0.001, *η*^2^ = 0.232; group × time: *F* = 17.62, *p* < 0.001, *η*^2^ = 0.22; figure 2). The model including the group × time interaction (BF_10_ = 9.875 × 10^31^) was decisively better (BF_10_ = 5.14 × 10^20^) than a model which only included time (BF_10_ = 1.921 × 10^11^) so we can accept the hypothesis that the intervention shifted bias over time relative to the control.

### 3.3. Self-report outcome measures

**Figure 3.**
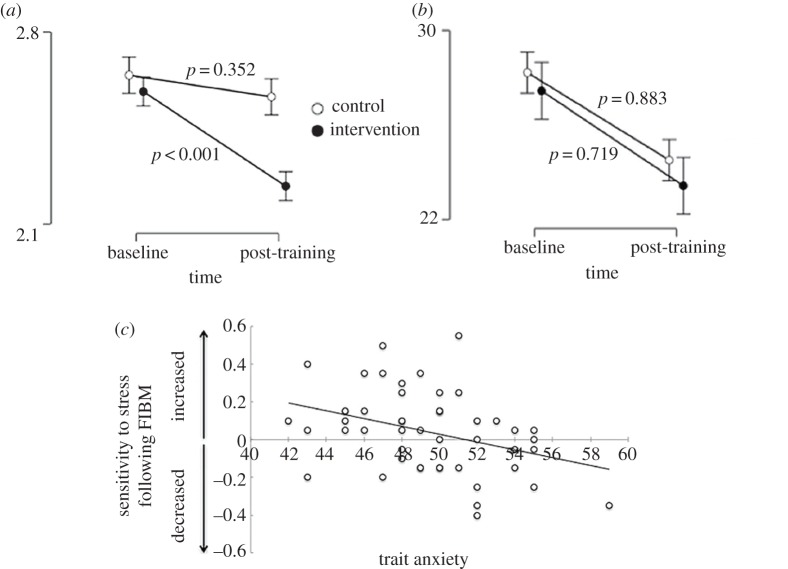
The simple effects across baseline and post-training for (*a*) Daily Stress Inventory (DSI) average impact rating. (*b*) DSI frequency measure. Error bars represent s.e.m. (*c*) Correlations between stress-SART threat-potentiated (threat condition minus safe condition) shift (time 2 minus time 1) and baseline trait anxiety in the FIBM group.

Scores (table 1*a*) did not differ substantially between groups over time for the BDI, STAI, PANAS or AIEO (see electronic supplementary material, table S1 for means and standard deviations). Bayes factor analysis revealed all models including the group × time interaction (barring the DSI (AIR)) to be worse than the model of time; we can, therefore, accept the null hypothesis that FIBM did not have an effect on the BDI, STAI, PANAS, AIEO or DSI (frequency). Notably, however, we did see clear evidence of a group × time interaction in the DSI (AIR) (*p* = 0.028). Simple effects show that this was driven by a change over time in the intervention (*F*_1,101_ = 16.99, *p* < 0.001), but not control groups (*F*_1,101_ = 0.087, *p* = 0.352), which resulted in weak evidence of group differences at post-training (*F*_1,101_ = 3.25, *p* = 0.074) but no clear evidence of differences at baseline (*F*_1,101_ = 0.11, *p* = 0.744; figure 3). Moreover, there was no comparable effect on DSI (frequency), indicating that FIBM induced an interpretation bias, not a detection bias. However, the Bayesian analysis showed that this interaction effect was only marginally better than a model with a main effect of time (BF = 1.01), so this is an inconclusive effect that warrants replication.

**Table 1.** (*a*) Frequentist and Bayesian models of self-report measures. Reported are the Beck Depression Inventory II (BDI-II), State-Trait Anxiety Inventory (STAI), Positive and Negative Affect Scale (PANAS), Daily Stress Inventory (DSI) and Ambiguous Interpretation of Emotional Outcomes (AIEO) between groups. DSI average impact rating (AIR) refers to sum of the stress ratings attributed to these events divided by the frequency. DSI (frequency) refers to the number of events reported to have occurred in the past 24 h. (*b*) Frequentist and Bayesian models of cognitive measures reported are the emotional dot-probe threat bias scores (the difference in mean reaction time (RT) and accuracy to negative versus neutral stimuli priming), stress-SART accuracy to ‘no-go’ stimuli and the proportion of hard trials chosen in the effort-expenditure for rewards task (EEfRT) between groups. BF, Bayes factor.

measure	*F* (time)	*F* (group × time)	effect size	BF_10_ (time)	BF_10_ (group × time)	BF (group × time)/time
(*a*) *Self-report measures*						
BDI-II	*F* = 12.232, *p* < 0.001	*F* = 1.383, *p* = 0.242	*η*^2^ = 0.012	BF_10_ = 34.923	BF_10_ = 5.152	BF = 0.148
STAI (state anxiety)	*F* = 0.149, *p* = 0.701	*F* = 0.294, *p* = 0.589	*η*^2^ = 0.003	BF_10_ = 0.16	BF_10_ = 0.008	BF = 0.05
STAI (trait anxiety)	*F* = 0.761, *p* = 0.385	*F* = 3.072, *p* = 0.083	*η*^2^ = 0.029	BF_10_ = 0.227	BF_10_ = 0.046	BF = 0.203
PANAS (positive)	*F* = 0.625, *p* = 0.431	*F* = 0.563, *p* = 0.455	*η*^2^ = 0.006	BF_10_ = 0.198	BF_10_ = 0.014	BF = 0.071
PANAS (negative)	*F* = 2.4, *p* = 1.24	*F* = 0.294, *p* = 0.589	*η*^2^ = 0.003	BF_10_ = 0.447	BF_10_ = 0.029	BF = 0.065
DSI (AIR)	*F* = 12.705, *p* < 0.001	*F* = 4.999, *p* = 0.028	*η*^2^ = 0.042	BF_10_ = 36.363	BF_10_ = 39.306	BF = 1.081
DSI (frequency)	*F* = 13.432, *p* < 0.001	*F* = 0.022, *p* = 0.882	*η*^2^ = 0	BF_10_ = 61.958	BF_10_ = 4.503	BF = 0.073
AIEO (positive)	*F* = 98.258, *p* < 0.001	*F* = 0.135, *p* = 0.714	*η*^2^ = 0.001	BF_10_ = 5.615 × 10^10^	BF_10_ = 4.632 × 10^12^	BF = 0.083
AIEO (negative)	*F* = 21.024, *p* < 0.001	*F* = 0.577, *p* = 0.499	*η*^2^ = 0.005	BF_10_ = 1230.759	BF_10_ = 96.04	BF = 0.078
(*b*) *Cognitive measures*						
dot probe (RT)	*F* = 8.113, *p* = 0.005	*F* = 0.131, *p* = 0.719	*η*^2^ = 0.001	BF_10_ = 8.152	BF_10_ = 0.348	BF = 0.043
dot probe (accuracy)	*F* = 0.197, *p* = 0.658	*F* = 1.464, *p* = 0.229	*η*^2^ = 0.015	BF_10_ = 0.167	BF_10_ = 0.035	BF = 0.21
Stress-SART	*F* = 0.186, *p* = 0.667	*F* = 1.415, *p* = 0.237	*η*^2^ = 0.014	BF_10_ = 0.125	BF_10_ = 1.823	BF = 14.584^a^
EEfRT	*F* = 23.063, *p* < 0.001	*F* = 3.655, *p* = 0.059	*η*^2^ = 0.029	BF_10_ = 2251.673	BF_10_ = 974.401	BF = 0.433

^a^This strong BF value is a reflection of this task showing no main effect of time, rather than a strong group × time interaction which, at BF_10_ 1.823, is a weak model.

### 3.4. Cognitive processing outcome measures

As highlighted in table 1*b*, scores did not differ substantially between groups over time for the dot probe, stress-SART or EEfRT (see electronic supplementary material, table S1 for means and standard deviations). However, there was weak evidence in frequentist analyses of the EEfRT data. Simple effects show that this was driven by changes in both groups over time, albeit with weaker evidence for change in the intervention group (control: *F*_1,101_ = 22.76, *p* < 0.001; intervention: *F*_1,101_ = 4.14, *p* = 0.045). There was also a numerically smaller decrease in the proportion of hard trials (versus easy trials) chosen by the intervention group (group differences not significant at baseline (*F*_1,101_ = 0.02, *p* = 0.88) or post-training (*F*_1,101_ = 0.9, *p* = 0.344)). Bayes factor analysis revealed the time model as the winning model, however, this was only 2.31 times better than the group × time interaction model indicating no definitive evidence in favour of either model.

*Exploratory post hoc analysis:* The effect of baseline trait anxiety and BDI on the omnibus interaction or FIBM across training for the intervention group, and the effect of baseline balance point and the omnibus interaction for the intervention group.

As participants were recruited from a healthy sample, it was possible that those in the intervention group already had a positive bias and so FIBM could not push them further. In order to see whether the effect of the manipulation was modulated by individual differences in baseline depression or anxiety symptoms, baseline BDI (intervention: *M* = 9.08, s.d. = 7.73, range = 30; control: *M* = 9, s.d. = 7.99, range = 34) and trait anxiety (intervention: *M* = 49.31, s.d. = 3.69, range = 17; control: *M* = 50.48, s.d. = 4.2, range = 18) were added as a covariate to omnibus interactions of interest for the FIBM group and then broken down into constituent correlations. There was strong evidence that the effect of the manipulation interacted with trait anxiety for the stress-SART (time × threat × trait anxiety interaction, *F*_1,48_ = 7.322, *p* = 0.009). This was driven by reduced stress-potentiated inhibition following FIBM only in those with higher trait anxiety (figure 3*c*, *r* = −0.365, *N* = 52, *p* = 0.008). This suggests that FIBM only reduced cognitive measures of negative affect in those with higher levels of trait anxiety (indeed, it may have had the opposite effect in those with low trait anxiety), providing evidence that these results were moderated by trait anxiety. There was no clear evidence of an interaction between the BDI and any outcome measure (all *p* > 0.229).

In a second post hoc analysis, we explored possible correlations between the first acquired balance point and the manipulation on our effects of interest (calculated as a baseline subtracted from post-training contrast). There was a marginally non-significant correlation between the balance point at baseline and state anxiety ratings over time (*r* = −0.272, *N* = 52, *p* = 0.051) in the intervention group, whereby those with a lower balance point at baseline (i.e. higher negative bias) showed a greater reduction in state anxiety post-training. This was not shown in the sham group (*r* = 0.011, *N* = 52, *p* = 0.942); however, the difference between the intervention and sham slopes was not significant (*p* = 0.149). There was no time × BP interaction in any of the other variables of interest (all *p* > 0.115).

## 4. Discussion

This study sought to (i) validate a novel FIBM paradigm, as well as (ii) explore transfer effects to self-report measures and cognitive processes in a healthy sample. In accordance with our first hypothesis, FIBM successfully induced (and replicated) a substantial positive bias in the intervention group, alongside no significant change in the control group. However, counter to our hypotheses, FIBM failed to transfer to the majority of outcome measures. Indeed, Bayesian analyses enabled us to accept the null hypothesis that FIBM had no effect on most measures. Nevertheless, for two measures, one self-report measure of stress impact and one cognitive measure of anhedonia, FIBM showed inconclusive effects that were in the predicted direction.

FIBM did not influence most self-report measures (BDI, STAI, PANAS, DSI (frequency) or AIEO score) at follow-up. This contrasts with a number of previous findings showing CBM-induced changes in mood using different techniques (e.g. [25,26,52]), though is similar to meta-analytic results considering alternative CBM tasks [31–33]. There was weak evidence of omnibus interaction for the DSI AIR measure, suggesting that post-FIBM daily events were perceived as less stressful. While it should be noted that this effect would not withstand correction for the multiple comparisons used in this paper, the Bayesian analyses, for which multiple comparison correction is less of an issue, do nevertheless provide weak evidence in favour of this effect. There was also some evidence for a correlation between the first acquired FIBM balance points and state anxiety over time, indicating that those in the intervention group with a lower balance point at baseline (i.e. higher negative bias) showed a greater reduction in state anxiety post-training (which was not shown in the sham group). This provides some evidence for the efficacy of the paradigm; however, the difference between the intervention and sham slopes was not significant, so strong inferences should not be drawn.

The dot probe is a common paradigm in CBM research [3], but transfer effects were not apparent in this study. Moreover, the dot probe showed a significant main effect of time indicating poor reliability over multiple testing sessions (e.g. [53]). This unreliability might plausibly explain the mixed positive and null FIBM findings using the dot-probe paradigm with repeated measures designs. In future, exploring alternative outcomes for the dot probe which have better reliability than traditional scores may be useful (e.g. calculating trial level bias scores [54]). In line with well-replicated findings, we found a difference in accuracy while under threat versus safe in the stress-SART, though this did not also differ between groups. The EEfRT has previously shown differences between individuals with anhedonia and healthy controls [36] and although there was a hint towards a similar effect of decreased anhedonia post-FIBM, this was seen alongside changes in both groups over time and evidence for the effect was inconclusive.

This proof of concept study only considered effects in healthy individuals. Although, as such individuals are clearly amenable to training, and may indeed possess sub-threshold symptoms, we also explored the possibility that results were moderated by symptoms in some individuals. An interaction with baseline trait anxiety was seen in the Stress-SART, indicating that transfer effects were observed in higher anxiety individuals only. The lack of a transfer effect to the Stress-SART may therefore be a result of the healthy sample used in this study. Notably this task was not among those in which a weak effect was observed, although the clear shift in biases on the training task suggests that our whole sample was amenable to FIBM. Future work in clinical populations will help disambiguate this. In fact, a reasonable parallel to the effects observed in this study may be working memory training, which does not transfer well to untrained cognitive operations in healthy samples [55], but shows promise as a tool for general cognitive improvement in impaired populations (e.g. [56,57]).

Further research is also needed to clarify the tentative self-report stress and cognitive effort effects observed here. One possibility, for example, is that this paper is underpowered to detect transfer effects. We powered the study for 50% of the effect size of the modification effect, and indeed, comprehensively replicated this effect. However, any effect size of *transference* to both cognitive and self-report measures is unknown. Given the complexity of any mechanism by which a computerized training task could shift perception of faces and then influence behaviour, it seems highly plausible that the transference effect size is considerably smaller than 50% of the training effect. As such, future work in a larger sample is warranted. One counter-argument to this is that if the effects are so small that they require large samples to detect then they are likely to be too small to be meaningful. However, at a population level even tiny effects can be meaningful (e.g. small effects in cancer intervention studies which may translate to meaningful impact at the population level [58]). Given the large impact and cost of mood disorders on the one hand, and the relatively low cost of providing FIBM training on the other, clarifying whether even small effects exist is probably worthwhile.

Another explanation for our lack of effects is that while one week was sufficient to induce a change in bias, it may not be long enough to observe corresponding changes in mood. For instance, improvements in mood may only occur as the individual learns how to interact with and respond to the world within the new, more positive cognitive framework [59] and this process takes longer than a week. However, counter to this argument is that Munafò *et al*. (2016, personal communication) showed no effects on some measures of mood at six-week follow-up. Although a substantial FIBM effect was observed, it is possible that training at home (versus in laboratory) may have affected the strength of effects. It may also be that the tasks selected in this study were too far removed from the process of disambiguating facial expressions. We chose these tasks, which have been shown to be disrupted in depression, precisely because they are so different. Specifically, if transfer of effects occurs then it should shift other processes that are sensitive to depression. However, perhaps tasks closer to the underlying process being targeted would be more successful. In fact, it may be that, as face perception is disrupted in social anxiety [18], we may have seen transfer to measures related to social anxiety as opposed to depression. Another possibility is that FIBM will not transfer to any of the measures because the balance point shift in the intervention group is driven by a generic learning effect. As training was executed by giving participants correct/incorrect feedback, it is possible that participants were simply learning the ‘correct’ answer rather than actually changing their interpretation. Our observed weak effects in the right direction argue against this possibility, as does prior evidence of transfer to unlearned faces [22], but it is nevertheless worth considering if these effects fail to replicate. Finally, it should be noted that this FIBM task has been shown to transfer to other tasks measuring facial interpretation [22]. We did not attempt to replicate this effect here, as we were interested in transfer to other, clinically meaningful tasks. However, in retrospect replication of this effect in this sample would have been a valuable positive control.

Overall, the results of this study suggest that the clinical potential of this FIBM technique should be approached with caution. Our study contributes to a body of findings that have failed to show clear positive effects of multi-session FIBM on symptoms of mood and anxiety disorders (e.g. [30,32,33]). Specifically, we add to these findings by largely showing no transfer effects to some measures of common self-reported mood disorder symptoms and a battery of cognitive processing measures, which have previously been associated with anhedonia [36] and stress response [44]. Although previous studies have been criticized for their failure to show effects due to a small effect of CBM [14], this study has shown a lack of transfer effects despite a substantial bias modification in a healthy sample. Unless the weak effects in this paper replicate in larger, clinical samples, we posit that the FIBM task considered may not be an efficacious intervention in affective disorders.

## Supplementary Material

CBM supplement; Figure 1; Figure 2; Figure 3

## Footnotes

1 Protocol for the current study: https://osf.io/65a4y/.

2 A demonstration version of the cognitive bias modification task can be seen at: https://bertmichael.firebaseapp.com/.

## Data accessibility

The data have been deposited in an external repository: https://osf.io/7uzwx/ [60].
