# Supplementary material for: Cognitive bias modification for facial interpretation: a randomized controlled trial of transfer to self-report and cognitive measures in a healthy sample
Source: R Soc Open Sci. 2017 Dec 13;4(12):170681. doi: 10.1098/rsos.170681 (PMC5749989; doi:10.1098/rsos.170681)
Supplement: CBM supplement; Figure 1; Figure 2; Figure 3 [file rsos170681supp1.docx]

*Figure 1*. CONSORT diagram of study procedure.


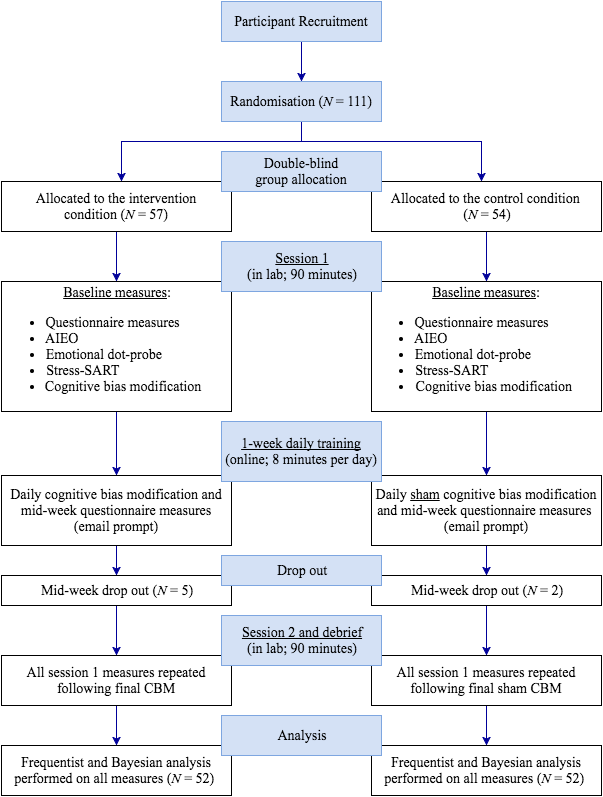


*Table 1*. Mean score (and standard deviation (SD)) of Daily Stress Inventory (DSI), Beck Depression Inventory (BDI), State-Trait Anxiety Inventory (STAI), and the Positive and Negative Affect Scale (PANAS), DSI frequency (the number of events reported to have occurred in the past 24 hours), DSI average impact rating (the sum of the stress ratings attributed to these events divided by the frequency), emotional dot probe threat bias scores (the difference in mean reaction time (RT) and accuracy to negative versus neutral stimuli priming), the proportion of hard trials chosen in the effort expenditure for rewards task (EEfRT) between groups, and stress-SART accuracy to “no-go” stimuli for safe and threat conditions.

| *Baseline, mean (SD)* | *Intervention (N=52)* | *Control (N=52)* |
| --- | --- | --- |
| BDI | 9.08 (7.73) | 9 (7.99) |
| State anxiety (STAI) | 49.5 (5.69) | 50.56 (4.11) |
| Trait anxiety (STAI) | 49.31 (3.69) | 50.48 (4.2) |
| Positive affect (PANAS) | 22.4 (5.51) | 21.27 (5.61) |
| Negative affect (PANAS)  DSI average impact rating  DSI frequency  Positive interpretation (AIEO)  Negative interpretation (AIEO)  Dot probe (reaction time)  Dot probe (accuracy)  EEfRT  Stress-SART (safe)  Stress-SART (threat)  2  *Post-training* | 22.15 (4.91)  2.58 (0.85)  27.63 (14.91)  9.15 (2.82)  4.54 (2.96)  22.38 (33.02)  -0.26 (3)  45.6% (0.17)  0.61 (0.2)  0.64 (0.2)  *(N=52)* | 23.13 (5.87)  2.64 (1)  27.88 (15.75)  9.77 (3.06)  4.69 (3.25)  22.25 (36.44)  0.65 (1.66)  46.1% (0.16)  0.62 (0.17)  0.68 (0.17)  *(N=52)* |
| BDI | 7.37 (8.02) | 7.9 (7.71) |
| State anxiety (STAI) | 49.69 (4.88) | 50 (6.11) |
| Trait anxiety (STAI) | 49.63 (4.09) | 48.96 (4.61) |
| Positive affect (PANAS) | 21.13 (5.33) | 21.25 (5.5) |
| Negative affect (PANAS)  DSI average impact rating  DSI frequency  Positive interpretation (AIEO)  Negative interpretation (AIEO)  Dot probe (reaction time)  Dot probe (accuracy)  EEfRT  Stress-SART (safe)  Stress-SART (threat) | 21.75 (4.83)  2.24 (0.8)  23.79 (14.63)  12.58 (4.4)  3.5 (3.05)  5.92 (43.53)  -0.06 (3.25)  42.2% (0.21)  0.58 (0.24)  0.66 (0.2) | 22.04 (6.36)  2.56 (1.02)  24.25 (16.07)  13.39 (4.29)  3.24 (3.14)  9.5 (45.17)  0.22 (1.8)  38% (0.21)  0.62 (0.21)  0.68 (0.18) |
